# Refractory Obstructive Sleep Apnea Secondary to Vagal Nerve Stimulation

**DOI:** 10.7759/cureus.67402

**Published:** 2024-08-21

**Authors:** Ryan P Coburn, Melissa C Lipford

**Affiliations:** 1 Department of Neurology, Mayo Clinic, Rochester, USA; 2 Department of Neurology, Division of Pulmonary and Critical Care, Center for Sleep Medicine, Mayo Clinic, Rochester, USA

**Keywords:** obstructive sleep apnea, worsening obstructive sleep apnea (osa), iatrogenic obstructive sleep apnea, vagal nerve stimulator, obstructive sleep apnea (osa)

## Abstract

Vagal nerve stimulator (VNS) devices are commonly used as a non-pharmacologic option for improved seizure control in patients with refractory epilepsy. However, a side effect associated with VNS device placement includes sleep-disordered breathing, which is complicated by the fact that a significant minority of patients with epilepsy have sleep-disordered breathing. We describe a patient with iatrogenically worsened refractory obstructive sleep apnea (OSA) secondary to VNS device placement, which resolved upon turning off the VNS device. This case highlights the need to screen for OSA in patients who are candidates for VNS device placement, as iatrogenic sleep-disordered breathing could place the patient at risk for adverse clinical outcomes, as well as paradoxically worsen seizure control due to poor quality sleep.

## Introduction

Drug-resistant epilepsy (DRE) poses a significant challenge for providers, with an estimated one-third of patients with inadequate seizure control despite two anti-seizure medications (ASMs) at therapeutic doses [1]. Additional medications are unlikely to lead to seizure freedom and may instead result in medication side effects [2]. With additional ASMs unlikely to be effective, non-pharmacologic options for seizure control are considered in the Neurologist’s armamentarium. Such options include the ketogenic or low glycemic index diet, surgical intervention such as resection of a structural lesion, or a neurostimulatory method such as a vagal nerve stimulator (VNS) [3].

The mechanism by which vagal nerve stimulation reduces seizure burden is not yet well elucidated. Proposed mechanisms include release of inhibitory neurotransmitters, desynchronization of cortical hypersynchronization during seizure activity, and increased blood flow to limbic and cortical brain regions, among others [4].

Side effects related to vagal stimulation commonly include hoarse voice, cough, and paresthesia [5]. More significant side effects are related to cardiovagal function; thus, cardiac conduction disorders serve as a contraindication to VNS placement, as increased vagal efferent firing could lead to symptomatic arrhythmia. A relative contraindication to placement includes a history of obstructive sleep apnea (OSA), as VNS devices have been shown to worsen or even cause iatrogenic obstructive and central sleep apnea [6]. 

We present a case of a woman with intractable epilepsy who upon VNS placement developed OSA which was refractory to continuous positive airway pressure (CPAP) and completely abated with the VNS device turned off.

## Case presentation

The patient is a left-handed female in her 60s with relevant comorbidities that include intellectual disability, severe OSA, and intractable multifocal epilepsy. 

The patient was diagnosed with epilepsy at the age of six months which was felt to be due to multiple intracranial vascular malformations. She had multiple seizure semiologies, including generalized tonic clonic, tonic, and atonic. Many ASMs were trialed at varying doses and combinations, including lamotrigine, carbamazepine, phenobarbital, felbamate, topiramate, valproate, and levetiracetam, with continued unacceptably high seizure burden. She underwent a partial corpus callosotomy at the age of 38. 

OSA was initially diagnosed at the age of 39. The patient had symptoms of daytime hypersomnolence and observed apneas in sleep, prompting further evaluation. A polysomnography study demonstrated very severe OSA with an apnea-hypopnea index (AHI) of 83 per hour (83/h). Disordered breathing events were eliminated by CPAP with a pressure of 9 cm water (H_2_O). 

Several years later, the patient underwent VNS implantation at the age of 49 due to continued high seizure burden. She experienced significant reduction in seizure frequency with VNS settings of current 1.5 milliamperes (mA), on time of 30 seconds, off time of 1.1 minutes, signal frequency of 25 Hertz (Hz), and pulse width of 250 micro-seconds (µsec).

**Figure 1:**
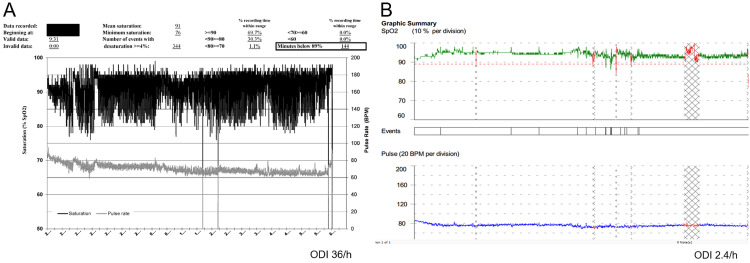
Patient Overnight Oximetry The patient’s overnight oximetry studies before (A) and after (B) the VNS stimulator was turned off are shown.  In (A), time is represented on the x-axis, with each dash representing 15 minutes.  Oxygen saturation and pulse are located on the y-axis (left and right side of the axis, respectively), with oxygen saturation in black and pulse in light grey.  In (B), oxygen saturation is located on the y-axis of the top graph and pulse is located on the y-axis of the bottom graph. The initial study (A) showed an oxygen desaturation index of 36/h, with the subsequent study once the VNS device was turned off (B) revealing a marked improvement to 2.4/h.  The figure was used with permission of Mayo Foundation for Medical Education and Research. All rights reserved. VNS: Vagal nerve stimulator; ODI: oxygen desaturation index

**Figure 2:**
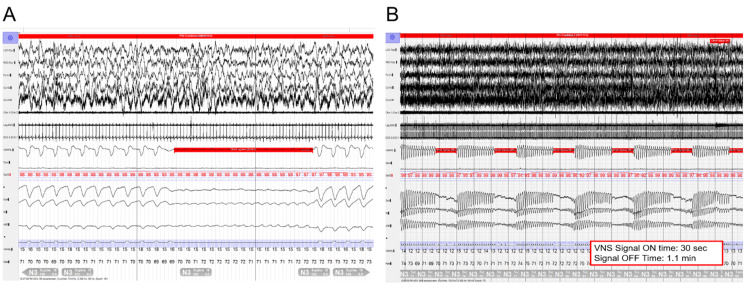
Patient Polysomnogram The patient’s polysomnogram prior to having their VNS turned off is shown.  In (A), an apneic event is seen, signified by the red highlighted portion of the study.  In (B), time is further compressed on the x-axis.  This allows for the ability to see multiple apneic events, again highlighted in red, which correspond to the VNS signal “on” time.  Return of respirations is seen during the “off” time.  The figure was used with permission of Mayo Foundation for Medical Education and Research. All rights reserved. VNS: Vagal nerve stimulator

She returned to the sleep clinic four years after VNS placement in the setting of losing her CPAP device and gaining 30 pounds. She was restarted on auto-titrating CPAP with a pressure window of 6-12 cm H_2_O. At a follow-up appointment, her CPAP device indicated persistent OSA, despite regular usage of CPAP. The estimated residual AHI was 20.2/h. Her auto-titrating settings were subsequently increased to 11-20 cm H_2_0, and she was asked to return with an overnight oximetry study performed on CPAP (Figure 1A). This study showed an oxygen desaturation index of 36/h (suggesting severe residual OSA). This was followed by overnight polysomnography (Figure 2A) which confirmed persistent OSA that did not respond to increasing CPAP pressures.

The fixed timing of her obstructive events matched with her VNS on cycle time, thus demonstrating the VNS device activating at the start of each obstructive apnea (Figure 2B). In consultation with her epilepsy physician, the VNS device was turned off overnight and a repeat overnight oximetry study was performed on CPAP (Figure 1B). This demonstrated a normal oxygen desaturation index of 2.4/h, indicating resolution of OSA.

## Discussion

The case we describe is an example of a patient with severe OSA previously well controlled with use of CPAP, who developed CPAP-refractory OSA following implantation of the VNS device. 

VNS device placement has been associated with worsening of underlying OSA and even de novo iatrogenic sleep-disordered breathing [7-9]. As available literature is predominantly small case series and case reports, true prevalence of sleep-disordered breathing following VNS placement is uncertain, but one meta-analysis estimates 31.9% in the adult population [10]. Apneas and hypopneas, as seen in our patient, are more frequent during VNS activation. 

Several mechanisms by which vagal nerve stimulation causes OSA have been proposed. Vagal afferent stimulation activates the dorsal motor nucleus of the vagal nerve, altering neuromuscular transmission to airway muscles of the pharynx and larynx, leading to pharyngeal closure. An alternative explanation is stimulation of vagal afferent projections to brainstem respiratory centers may influence breathing patterns [11]. The mechanism underlying vagal nerve stimulation and central sleep apnea is poorly elucidated [12]. 

An estimated 33.4% of patients with epilepsy have comorbid OSA [13]. As sleep is a well-recognized modifiable risk factor in seizure control, there is significant importance in treatment of underlying OSA, with positive pressure therapy shown to reduce seizure frequency over time [14]. Thus, the interplay between VNS placement and OSA represents a complicated dynamic. Screening for OSA before and after VNS placement should therefore be considered. 

The management of OSA following VNS placement should be managed in a multidisciplinary manner, with Neurology and Sleep Medicine providers optimizing VNS and CPAP settings, respectively. Adjustments to VNS settings to mitigate this effect include reducing stimulus intensity or frequency, decreasing pulse width, or increasing time off [12]. A programmable device that allows adjustments of the above settings during a specific time of day, or turning the device off overnight, represents an additional option. Trials of higher CPAP pressures may be necessary. However, as shown in our case, apneas may remain refractory to CPAP therapy. 

## Conclusions

We describe a case of CPAP-refractory OSA due to VNS device placement. This case highlights the importance of screening for OSA both before and after device placement, as proper management of OSA and improved sleep quality play an important role in seizure control. Adjustments to VNS settings or titration of CPAP pressures may be necessary, although some cases may remain refractory, and alternative solutions such as a programmable device may be required.

## References

[1] Kwan P, Arzimanoglou A, Berg AT (2010). Definition of drug resistant epilepsy: consensus proposal by the ad hoc Task Force of the ILAE Commission on Therapeutic Strategies. Epilepsia.

[2] Chen Z, Brodie MJ, Liew D, Kwan P (2018). Treatment outcomes in patients with newly diagnosed epilepsy treated with established and new antiepileptic drugs: a 30-year longitudinal cohort study. JAMA Neurol.

[3] Saxena VS, Nadkarni VV (2011). Nonpharmacological treatment of epilepsy. Ann Indian Acad Neurol.

[4] Di Lazzaro V, Oliviero A, Pilato F (2004). Effects of vagus nerve stimulation on cortical excitability in epileptic patients. Neurology.

[5] Ben-Menachem E (2001). Vagus nerve stimulation, side effects, and long-term safety. J Clin Neurophysiol.

[6] Toffa DH, Touma L, El Meskine T, Bouthillier A, Nguyen DK (2020). Learnings from 30 years of reported efficacy and safety of vagus nerve stimulation (VNS) for epilepsy treatment: a critical review. Seizure.

[7] Garrett AL, Burch J, Zhang Y, Li H, Sundar KM, Farney RJ (2023). Depicting and defining sleep disturbed breathing associated with vagal nerve stimulation. Sleep Med.

[8] Marzec M, Edwards J, Sagher O, Fromes G, Malow BA (2003). Effects of vagus nerve stimulation on sleep-related breathing in epilepsy patients. Epilepsia.

[9] Ebben MR, Sethi NK, Conte M, Pollak CP, Labar D (2008). Vagus nerve stimulation, sleep apnea, and CPAP titration. J Clin Sleep Med.

[10] Saleem M, Santhumayor B, Hasan E, Kolesnik M, Bernbaum M (2023). 0541 obstructive sleep apnea following vagus nerve stimulator implantation: a meta-analysis. Sleep.

[11] Malow BA, Edwards J, Marzec M, Sagher O, Fromes G (2000). Effects of vagus nerve stimulation on respiration during sleep: a pilot study. Neurology.

[12] Gurung P, Nene Y, Sivaraman M (2020). Vagus nerve stimulator (VNS)-induced severe obstructive sleep apnea which resolved after the VNS was turned off. Cureus.

[13] Lin Z, Si Q, Xiaoyi Z (2017). Obstructive sleep apnoea in patients with epilepsy: a meta-analysis. Sleep Breath.

[14] Pornsriniyom D, Kim Hw, Bena J, Andrews ND, Moul D, Foldvary-Schaefer N (2014). Effect of positive airway pressure therapy on seizure control in patients with epilepsy and obstructive sleep apnea. Epilepsy Behav.

